# Nanodomained Nickel Unite Nanocrystal Strength with Coarse-Grain Ductility

**DOI:** 10.1038/srep11728

**Published:** 2015-06-30

**Authors:** Xiaolei Wu, Fuping Yuan, Muxin Yang, Ping Jiang, Chuanxin Zhang, Liu Chen, Yueguang Wei, Evan Ma

**Affiliations:** 1 State Key Laboratory of Nonlinear Mechanics, Institute of Mechanics, Chinese Academy of Sciences, Beijing 100190, China; 2 Department of Materials Science and Engineering, The Johns Hopkins University, Baltimore, Maryland 21218, USA

## Abstract

Conventional metals are routinely hardened by grain refinement or by cold working with the expense of their ductility. Recent nanostructuring strategies have attempted to evade this strength versus ductility trade-off, but the paradox persists. It has never been possible to combine the strength reachable in nanocrystalline metals with the large uniform tensile elongation characteristic of coarse-grained metals. Here a defect engineering strategy on the nanoscale is architected to approach this ultimate combination. For Nickel, spread-out nanoscale domains (average 7 nm in diameter) were produced during electrodeposition, occupying only ~2.4% of the total volume. Yet the resulting Ni achieves a yield strength approaching 1.3 GPa, on par with the strength for nanocrystalline Ni with uniform grains. Simultaneously, the material exhibits a uniform elongation as large as ~30%, at the same level of ductile face-centered-cubic metals. Electron microscopy observations and molecular dynamics simulations demonstrate that the nanoscale domains effectively block dislocations, akin to the role of precipitates for Orowan hardening. In the meantime, the abundant domain boundaries provide dislocation sources and trapping sites of running dislocations for dislocation multiplication, and the ample space in the grain interior allows dislocation storage; a pronounced strain-hardening rate is therefore sustained to enable large uniform elongation.

## The dilemma of strength-ductility trade-off

Making materials both strong and ductile has been the pursuit of materials scientists and engineers for centuries. This is in fact a highly challenging proposition because strength and ductility are in general mutually exclusive^1^,^2^,^3^,^4^,^5^,^6^,^7^,^8^,^9^,^10^,^11^,^12^,^13^,^14^,^15^,^16^. For example, the yield strength (σ_y_) of a metal can be increased by refining its internal grain structure: extensive research for the past three decades has produced numerous ultrafine-grained (UFG)^3^,^5^,^6^,^7^,^8^,^9^,^10^,^11^ and nanocrystalline (NC)^4^,^17^,^18^,^19^,^20^,^21^,^22^ metals, which can have σ_y_ many times of that of coarse-grained (CG) counterpart due to the well-known effect of grain boundary (GB) strengthening. But their elevated σ_y_ comes at the expense of ductility. In particular, the large uniform tensile elongation, a hall-mark advantage of conventional ductile metals, becomes disappointingly low. This is because the structural refinement takes away the room to store dislocations^3^,^11^,^12^,^13^, as the latter readily annihilate into the now-abundant GBs. The high density of dislocation sinks nullifies the dominant mechanism for strain hardening in metals. As a result, non-uniform deformation sets in soon after yielding starts.

The other most common way to strengthen a metal is “cold work”^12^,^13^,^14^,^15^. Via plastic deformation, dislocations multiply inside the material, which serve as obstacles against subsequent dislocation movements, elevating the stress needed to sustain plastic flow. However, the pre-worked metal has used up its capability to accumulate dislocations. The result is again a diminished strain hardening rate and hence the useful ductility (i.e., the uniform elongation before the onset of necking).

**Figure 1:**
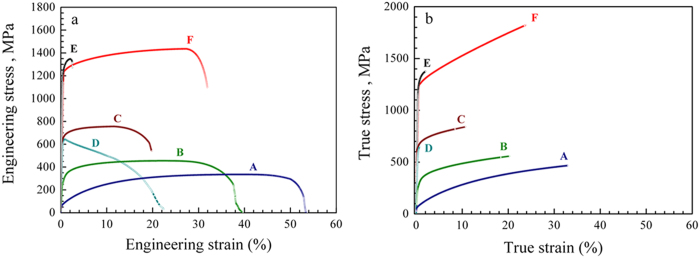
Tensile stress-strain curves of Ni. (**a**) Engineering stress-strain curves at a strain rate of 4 × 10^−4^ s^−1^: Curve A: as-annealed coarse-grained (CG) Ni with an average grain size (*d*) of 27 μm. Curve B: electrodeposited (ED) Ni (*d* = 1 μm). Curve C: electrodeposited ultrafine-grained (UFG) Ni (*d* = 200 nm); Curve D: UFG Ni obtained via equal channel angular pressing (ECAP) for one pass; Curve E: ED nanocrystalline Ni (*d*_*grain*_ = 18 nm); Curve F: electroplated nanodomained Ni (*d*_*grain*_ = 150 nm, *d*_*domain*_ = 7 nm). (**b**) True stress-strain curves, converted using standard equations (up to the maximum stress point where non-uniform elongation onsets) from the corresponding curves in (**a**). Note the simultaneous high strength and uniform elongation exhibited by nanodomained Ni, Curve F. See Supplemental Information for the gauge section dimensions of the samples.

**Figure 2:**
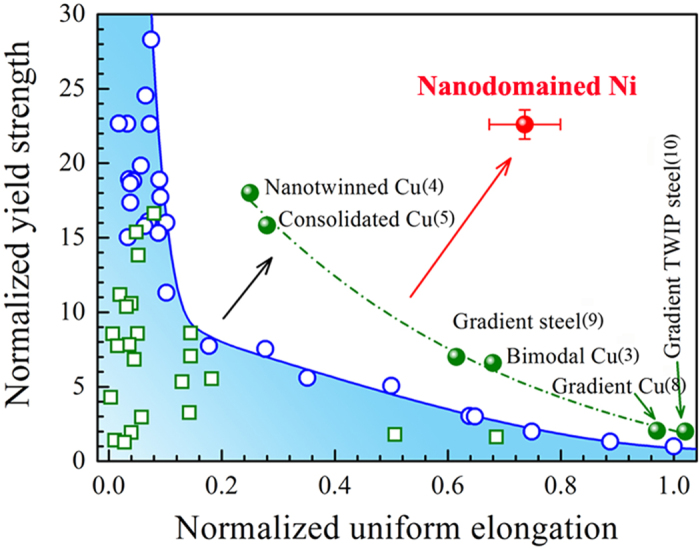
Normalized yield strength versus normalized tensile uniform elongation for nanostructured metals. Nanodomained Ni stands out as an exception “out of the box”, with its nanocrystal-level strength and coarse-grain-like ductility.

We use nickel (Ni) in this work to illustrate our points (see Methods and Figs S1 through S3 in Supporting Information (SI) about the experimental conditions used and the various forms of Ni prepared). In Fig. 1a, a typical tensile engineering stress-strain curve of an as-annealed CG Ni is shown as Curve A. Its mean yield strength is only 53 MPa, but the pronounced strain hardening capability due to dislocation accumulation delays necking to an elongation of the order of 40%. Typical electrodeposited (ED) Ni (Curve B and Curve C) has a higher strength but the uniform elongation decreases with decreasing grain diameter, *d*_*grain*_. Ni processed via severe plastic deformation (SPD)^12^,^13^,^14^,^15^ loses ductility even faster, see Curve D in Fig. 1a for Ni prepared via equal channel angular pressing (ECAP). In this latter case the UFG (*d*_*grain*_ is a few hundreds of nanometers) structure drives the σ_y_ up to ~600 MPa, but the uniform elongation decreases to one percent. Also included in Fig. 1a is Curve E for NC Ni (*d*_*grain*_ = 18 nm)^17^,^18^,^19^,^20^,^21^,^22^. Now the σ_y_ is ~1.3 GPa, but the uniform elongation is also limited to a couple of percent. Figure 2 summaries such a strength-ductility trade-off for different types of Ni and Cu (the blue region; similar trends^11^,^12^,^13^,^14^ are well known for other metals as well).

The internal dislocation structure is the root cause of the behavior above. TEM images of the ED-Ni (Fig. S3a) and ECAP-Ni (Fig. S3b), examined post-mortem after tensile straining, are displayed in SI. As expected from prior experience with such UFG grain structures, dislocation storage is limited inside the Ni grains (Fig. S3a): tilting the sample over an extensive range in the TEM did not detect strong diffraction contrast from dislocations. The exception is at the GBs, where dislocation sources reside. During plastic deformation the majority of the dislocations run across the small grain to annihilate inside the GBs on the opposite side, leaving behind little dislocation debris/tangles in the grain interiors. In the SPD case (Fig. S3b), most GBs themselves evolve from the rearrangements of deformation saturated dislocations^11^,^12^. The capability to store dislocations has been largely exhausted during SPD. The low strain hardening capability leading to early termination of uniform elongation is well known for UFG and NC metals^11^,^12^,^13^,^14^,^15^,^16^.

## Our new strategy: defect engineering employing nanodomains

Driven by the need to retain ductility while reaping the strengthening benefits of nanostructuring, several strategies have been reported in recent years to elevate strength without losing the strain hardening capability^3^,^4^,^5^,^6^,^7^,^8^,^9^,^10^,^11^. In Fig. 2, we cite a few previously reported successful attempts as examples^3^,^4^,^8^,^9^,^10^. In this figure we use the normalized yield strength, (σ_*y,n*_), as well as the normalized uniform tensile elongation (ductility), ε_*n*_, both with the CG counterpart of the metal as the reference (any CG metal is at (1,1)). When the internal microstructure is refined, all the way to UFG and nanocrystalline (blue region), σ_*y,n*_ is elevated from 1 up to ~25, but the ε_*n*_ is much reduced and typically down to <0.1 for NC metals. The black arrow points to several recent successes (green solid circles) in jumping out of this blue region. For example, on the high strength end, nanotwins can strengthen Cu to σ_*y,n*_ ~18 (similar to NC Cu), and its ε_*n*_ is much better than NC Cu^4^. On the high ductility end, one can use a distribution of grain sizes: examples are the bimodal Cu^3^ or gradient structures^8^,^9^,^10^ for which the ε_*n*_ is retained at >0.7, not far below the CG counterpart. However, these cases still fall under a compromising trade-off, it is just that the “banana curve” (green dashed line) is shifted upwards relative to the traditional one (blue region). Specifically, for nanotwinned Cu the ε_*n*_ is only ~0.2, far below CG, whereas for bimodal Cu, the σ_*y,n*_ is only elevated to ~7, nowhere close to NC Cu. The bottle neck problem is still a sacrifice of either the strength or the ductility, or both.

In other words, what remains unoccupied so far is a large area near the upper-right corner in Fig. 2, where the product of strength and ductility is maximized. Specifically, we want to harden the metal to strength well over 1 GPa, at the same level as that achieved with contiguous NC grains (Curve E in Fig. 1a). So a sufficiently high density of defects (GBs and dislocations), beyond that in UFG microstructures, needs to be planted in the material to block running dislocations. In the meantime, we must also be able to effectively store dislocations to impart pronounced strain hardening in subsequent tensile deformation, to the extent that the uniform tensile elongation is not much compromised with respect to that of CG metals. For that to happen, the distribution of pre-planted defects has to be engineered in such a way that they provide numerous sources and traps of dislocations while still leaving ample space to allow for the multiplying dislocations to accumulate efficiently. This is not possible with the contiguous NC grains everywhere in the material, because as mentioned earlier the high-density high-angle GBs constitute effective sinks that suck away almost all the dislocations running in the tiny grains. The challenge, therefore, is to design and manufacture defect structures that can serve dual purposes in a metal.

To this end, we have conceived a new strategy. The idea is to architect a spread-out distribution of nanoscale domains that constitute only a few % of the volume of the UFG grains. This may seem to be akin to “precipitation hardening” in alloys, but an obvious difference is that in our case we have a single-phase material (the domains are elemental Ni as well, rather than second phase particles). The domains dispersed in the matrix will be made only a few nanometers in diameter, but numerous in populations (hence close spacing). Running dislocations will then inevitably hit these domains and be blocked. This is because i) domains on this size scale will be difficult for dislocations to operate inside them (akin to NC grains) or cut through them (akin to precipitates in alloys), and ii) the incident lattice dislocations would react with the dislocations in the domain boundaries to form immobile junctions^23^,^24^,^25^,^26^,^27^,^28^. At a spacing of a few tens of nanometers, the nanodomains would elevate the strength to GPa level (see the calculation of Orowan-type strengthening in SI). In the meantime, the domain boundaries can emit, and are pinning sites of, dislocations, giving the latter more chances to interact, multiply, and tangle up. This leads to more and more carriers that are “in each others’ way” to make their movement increasingly difficult. As the nanodomains occupy only a few % of the total volume, plenty of room is “reserved” in the grain interior for dislocation lines to lengthen and accumulate.

**Figure 3:**
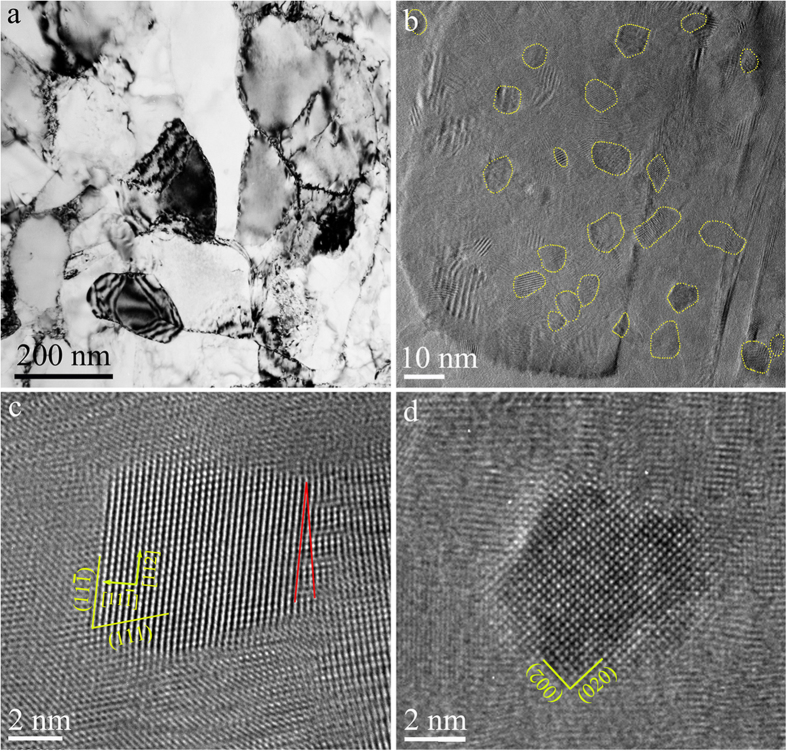
TEM images of nanodomained Ni. (**a**) Bright-field TEM micrograph showing the UFG grains with nanoscale domains. The majority of nanodomains inside the grains have low-angle (<15° misorientation angle) domain boundaries, making it difficult to tell them apart from the matrix due to weak diffraction and phase contrast. HREM was therefore used to reveal the presence of nanoscale domains: with [011] zone axis, the {111} planes in the domain deviate by a few degrees (see distribution in Fig. S5 in Supplementary information) relative to those in the matrix. The nanoscale domains (circled by yellow dotted lines in a grain) spread-out in the UFG grains are shown in (**b**). The size distribution of nanodomains is presented in Fig. S4 in Supplementary information. About 95% of the ~300 domains examined have low-angle domain boundaries (LADBs); one close-up view is shown in the HREM image in (**c**) the misorientation angle between (111) planes is marked by two straight lines in red. An example of the occasional domains with high-angle domain boundaries (HADBs) is given in (**d**).

Such a microstructure can be achieved during the growth process of a metal. In other words, the “defects” can be sowed into the grains via purposely-designed excursions of deposition/growth parameters (see details in Methods). For Ni, we have developed a pulsed electrodeposition procedure to produce nanodomains: the disturbance from high current density pulses, together with the supply of grain refining agent, initiate numerous nanoscale domains, which are crystallographically misoriented by a few degrees with respect to the matrix grain. These domains do not become contiguous, as their growth is interrupted, due to the ensuing disturbance as the plating current is quickly ramped down to a level that grows UFG grains. As a result, nanoscale domains intermittently emerge inside larger grains. See the Methods section for details of the processing protocol. In conventional bright-field TEM images, such as that in Fig. 3a (and Fig. S2), the domains are difficult to see, because most of them are tiny and have low-angle misorientations with respect to the matrix UFG grains. High-resolution TEM, however, reveals the presence of many domains ranging from 3 to 12 nm in size (with an average domain diameter, *d*_*domain*_, of ~7 nm and the volume fraction of ~2.4%, see size distribution and misorientation angle distribution plots before and after tensile straining in Figs S4 and S5). Examples are shown in the high-resolution images in Fig. 3b through 3d.

**Figure 4:**
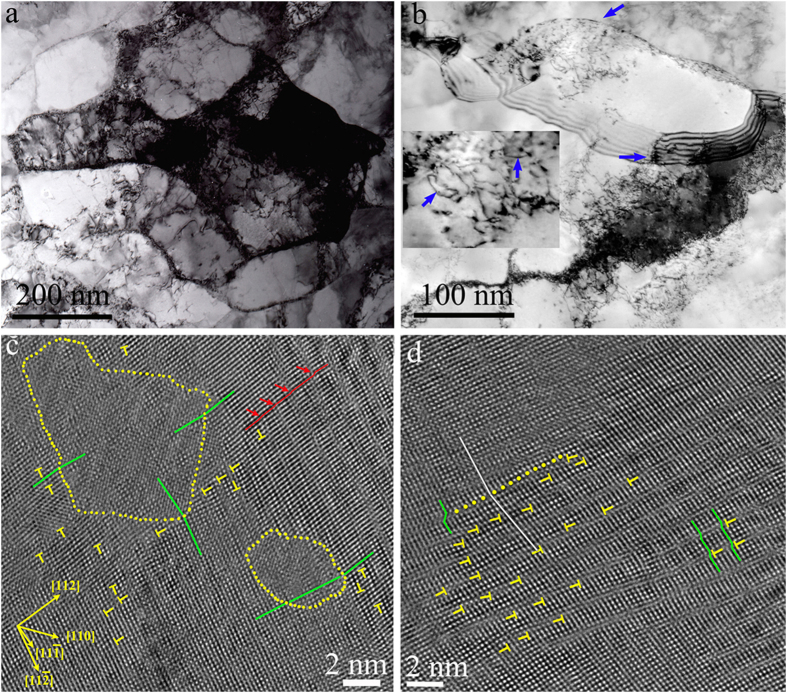
Dislocation accumulation in nanodomained Ni upon tensile deformation. (**a**) Bright-field TEM micrograph taken after tensile straining to 18% elongation, showing dislocations of high density that are tangled inside the grains. In (**b**) dislocations emitted from GBs (blue arrows) interact and accumulate in large numbers inside the grains. In the inset, the bowing red arrows mark the dislocations with ends pinned by nanodomains. HREM images are shown in (**c**) and (**d**) where the nanodomains are delineated by yellow dots. The green lines indicate misorientations ranging from 2° to 6° across the domain boundaries. In (**c**) the 2D lattice fringes in the domains are ill-defined as compared with the neighboring matrix area. Stacking faults are marked with red arrows. Note that dislocation density (marked by T, Burgers vector *b* = 1/2[011]) is considerably higher near the nanodomains than that away from the nanodomains. The dislocation pile-ups against a domain boundary are especially obvious in (**d**) there are few dislocations inside the nanodomain (upper-left corner), while many dislocations on {111} planes are blocked by the domain boundary on the other side.

The dislocation structure in such nanodomained Ni after tensile straining to 15 to 20% is shown in the post-mortem TEM images, Fig. 4a,b. In contrast to the case without nanoscale domains, now there are many dislocations stored inside the grains. The inset in Fig. 4b shows examples of bowing dislocations (marked by blue arrows), with ends pinned by nanodomains: the bulging dislocation lines in between nanodomains indicate that dislocations are pushed by the externally applied stresses to bow around the nanodomains. High-resolution images, Fig. 4c,d, confirm that the dislocation density is much higher in regions near the low-angle domain boundaries (LADBs) than those away from the domains. Some of these dislocations (full dislocations and partial dislocations) were emitted from these boundaries themselves, while many others came from other sources (at UFG GBs and/or boundaries of other domains). Several dislocation pile-ups at the domain boundary are clearly seen in Fig. 4d. Hence, the nanodomains act as strengthening agents (like precipitates and dispersions in alloys) to block dislocations, and at the same time emit dislocations that tangle with incoming dislocations. This multiplies and accumulates dislocations (see Fig. 4). As semi-quantitatively analyzed and shown in Fig. S6 and S7, the nanodomains thus serve the dual purposes of i) impeding the motion of dislocations (elevating strength) and ii) facilitating their interaction and accumulation during ensuing dislocation activities (resulting in strain hardening), “killing two birds with one stone”.

**Figure 5:**
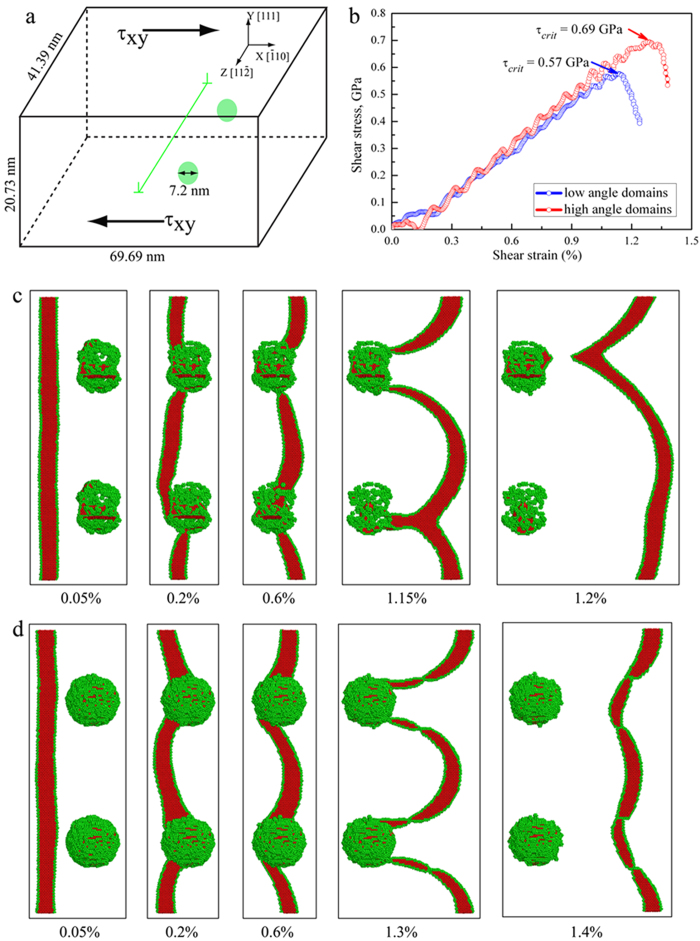
Molecular dynamics simulations of slip-nanodomain interactions. (**a**) Configuration for simulation cell with a straight edge dislocation and two nanodomains. (**b**) Simulated shear stress-shear strain curves as the straight dislocation is blocked by the nanodomains; the critical shear stress (τ_*crit*_) at which the dislocation depins from the nanodomains is indicated in the figure. (**c**) A sequence of snapshots at different shear strains showing the pinning of the dislocation and its subsequent bowing around the nanodomains with LADB. (**d**) A sequence of snapshots at different shear strains showing the pinning of the dislocation and its subsequent bowing around the nanodomains with HADB.

This scenario is supported by MD simulations. As shown in Fig. 5 (and the difference between high-angle and low-angle boundaries is shown in Fig. S8), nanodomains force the incident dislocation line to bow in between them, much like hard particles in precipitation hardening. Also, the boundaries act as active dislocation sources during deformation: the MD simulations in SI (see Fig. S9) demonstrate that some dislocations making up the domain boundaries split into partial dislocations that are emitted successively; at least two slip systems are seen to have been activated (in HREM observations as well, see Fig. 4c). The incoming lattice dislocations bulging in between nanodomains and the dislocations emitted from nanodomain boundaries entangle, and generate even more dislocations when they interact with one another. The accumulating dislocations in the grain volume (Figs 4 and 5) impede dislocation movements in subsequent deformation, and are hence responsible for enhanced strain hardening. To clearly demonstrate the pronounced strain hardening, we have converted the engineering stress-strain curves in Fig. 1a into true stress-true strain curves in Fig. 1b. The curve of the nanodomained Ni is fast ascending at a steady strain-hardening rate (slope) even at GPa-level flow stresses, allowing a tensile strength of nearly 2 GPa in true stress. With a combination of exceptional strength and ductility, the nanodomained Ni stands out in Fig. 2, well separated from all previous forms of Ni (from coarse-grained all the way to nanocrystalline) with a homogeneous structure.

The nanodomains, albeit very small, appear to be stable during tensile straining and after long-time sample storage (see stress-strain curves in Fig. S10 in SI). During the deformation (and sample storage), one could conjecture that the departure of some of the dislocations in the domain boundaries^29^ (a source of dislocation emission^30^), or rotation of the lattice to decrease the misorientation angles^31^, may eventually merge the domains with the matrix, reducing the population of nanodomains. However, low-angle GBs have relatively low energy and the driving force for their disintegration is not very high, such that small-angle GBs can survive long storage or deformation^32^. They in fact often contain dislocations that cannot easily leave the domain boundaries. Also, the boundaries are continuously hit by incident lattice dislocations that increase the dislocation content in/near the boundary. Such tangling and blocking interactions with incoming dislocations help pin down the boundary (the increase of threshold stress for a low-angle GB to de-pin from extrinsic dislocations has been predicted by Lim *et al.*^33^), and the dislocations arriving at a domain boundary and subsequent relaxation may even render the boundary mechanically stronger^34^). Moreover, GB sliding is not activated here, because the deformation temperature is too low (room temperature is below 0.17 melting temperature of Ni) to allow sufficient diffusional creep, and the low-angle boundaries are not amenable to GB sliding^35^.

## Simultaneous high strength and ductility

With the TEM and MD evidence in hand, we now proceed with a semi-quantitative analysis to rationalize the strength and ductility observed. In Fig. 1a, we observe that the strength of the nanodomained Ni is only slightly lower than that of NC-Ni, i.e., the Ni composed entirely of equi-axed grains with *d*_*grain*_ = 18 nm^15^,^18^,^22^. In other words, while we have “reserved” the majority of the space for dislocation accumulation (nanoscale domains occupy only a few percent of the grain volume), the defects planted in the grain interior are effective and adequate in elevating the strength to a level achievable with homogeneous NC grains. This can be understood as follows. As confirmed in MD simulations, Fig. 5, incident lattice dislocations coming from the GBs of UFG grains usually get stopped at the boundaries of the nanoscale domains: the segment hitting the nanodomain fails to “penetrate” through the boundary but instead gets pinned by the boundary. Nucleating a new dislocation to bulge into the narrow nanodomain space would require a very high stress due to a large curvature (small radius of curvature)^13^,^14^. This justifies our earlier premise that the nanoscale domains act as strong obstacles to dislocation movements (much like hard particles in precipitation hardening). From Fig. 5, our simulations suggests that nanodomains with either high-angle or low-angle boundaries can pin dislocations for strengthening, although the elevated strength for low-angle boundaries is slightly smaller (0.57 GPa vs. 0.69 GPa). The reason is that even for a low-angle boundary, it is composed of a net of dislocations waiting to tackle running lattice dislocations. The dislocation spacing in the boundary wall is only a couple of nanometers^31^. As a result, the incident dislocation has a high probability to react with some of the boundary dislocations to form an immobile product that locks down^23^,^24^,^25^. The stress needed to depin has recently been predicted to approach 1 GPa^23^,^24^,^25^ (also see Fig. 5). As a result, the dislocation line bows around the nanodomains (see Fig. 5 and inset in Fig. 4b), and the stress elevation due to line tension can be estimated from Orowan strengthening^36^ to be of the order of *Gb/L*, where *G* is the shear modulus (76 GPa), *b* is the Burgers vector (0.2489 nm) and *L* is the average spacing between the nanodomains (see Fig. S6). This estimate suggests a strength elevation of ~0.8 GPa, slightly higher than what is observed in Fig. 1a (~0.6 GPa). One reason is that the strengthening effect of the domain boundaries is not the same as that of the assumed “hard particles” (see Fig. 5 and Fig. S8).

To elevate the storage rate (stored dislocation density normalized by experienced plastic strain), the way to go is to increase the volume fraction and decrease the size of the particles^37^. The use of very small and large number of particles was advocated before, for precipitation hardened^38^,^39^,^40^ and dispersion-strengthened alloys^41^. Our Ni is the first case demonstrating that a similar route is in fact highly effective when using distributed nanoscale domains (see Fig. S7 for an estimated dislocation storage rate as a function of the volume fraction and the size of the traps). With the sluggish dislocation motion of pinning/de-pinning in Fig. 5, there will be a greater likelihood of dislocation interaction and multiplication, for the crystal to attain a high dislocation density during deformation (see Fig. 4). This makes dislocation motion harder and harder, giving rise to enhanced strain hardening. This is in stark contrast with the case of NC metals, for which both TEM observations^22^,^42^ and MD simulations^43^ have shown that almost all the dislocations leaving the GBs traverse the grains and annihilate into the opposite GBs (sinks), with little chance and space to be retained inside the tiny grains.

In summary, to evade the long-standing strength-ductility trade-off dilemma, we have implemented a new strategy to control the density and distribution of defects (dislocations) in the as-prepared metal. Specifically, we have developed an experimental electroplating protocol, to deploy only ~2 vol% of nanoscale domains that spread out inside Ni grains. The nanodomained Ni is as strong as nanocrystals, and at the same time as ductile as CG metals, a combination not achieved before: in Fig. 2 our new data point jumps “out of the box”, in an unprecedented property space towards the upper right corner (red arrow and red solid circle). The extraordinary strengthening is attributed to the blocking of dislocations by the nanoscale domains (their dislocation boundaries), which, while at a fairly low volume fraction, play the role of precipitates (used in Orowan strengthening of alloys) to tackle the running dislocations such that the strength is elevated to the level of NC-Ni. At the same time, these intentionally pre-planted domains (large population of dislocation defects) encourage multiplication mechanisms and yet leave ample room inside the grains for the dislocations to entangle and store, producing a pronounced strain hardening rate at GPa-level flow stresses. This sustains uniform elongation to a level typical of face-centered-cubic metals that have the reputation to be very ductile. The extraordinary product of strength and ductility sets the nanodomained Ni apart from all previous attempts, see Fig. 2. As such, the heterogeneously architected nanostructure opens a new avenue towards strengthening a metal (or a single-phase alloy) to giga-pascal levels without much sacrifice of CG uniform elongation.

## Methods

### Electrodeposited nanodomained Ni

An annealed and polished Cu sheet was used as the substrate. The anode was a pure nickel plate. The ratio of anode area to cathode area was 4:1. Ni was electrodeposited onto the Cu cathode using a pulse electro-deposition method. The aqueous sulfamate-based electrolyte consists of Ni(NH_2_SO_3_)_2_·4H_2_O(800–1000 gl^−1^), NiCl_2_·6H_2_O (50 gl^−1^), boric acid(50 gl^−1^), soluble saccharin (2.5 gl^−1^), lauryl sodium sulfate (0.2–0.4 gl^−1^) and a small amount of additives. The electrolyte was held at a pH level of 3.5–4 and ~45 °C. The pulsed plating used a repeating square wave with a width of 5–10 ms. 1,4-butenediol was supplied as a grain refiner into the electrolyte at ~1 gl^−1^, to help induce nanoscale domains. The half cycle with high current density (20–30 A/dm^2^) concurrent with the high surfactant concentration supplied triggers the wide-spread emergence of nanoscale domains; whereas during the other half cycle of low current density (1–5 A/dm^2^), the rest of the grains continue to grow larger and enclose the nanodomains. After reaching a thickness of about 120 to 150 μm, the Ni plate was removed mechanically from the Cu substrate. The tensile samples were prepared by polishing away the initial layer of ~10 μm in thickness next to the substrate.

The impurities in as-deposited Ni were analyzed using inductively coupled plasma atomic emission spectrometry (IRIS Intrepid ER/S) (for metallic impurities) and instrumental gas analysis (IGA) (for light elements H, C, S, N, and O) (Evans Analytical Group, LLC. NY, USA). Table S1 in Supplementary Information lists the concentrations of the main impurities (in mass parts per million). Two batches of as-electrodeposited Ni plates were analyzed. The differences in main impurity contents were negligible, indicative of repeatability and consistency in reproducing identical compositions.

### Homogeneous Nanocrystalline and fine-grained Ni via electrodeposition

Fully dense electrodeposited nanocrystalline (NC) Ni sheets were procured from Goodfellow Inc. The as-received foil was 150 μm thick, with 99.8% purity and a mean grain size of ~18 nm. This is the same batch of Ni as the one we have investigated previously^42^. The electrodeposited fine-grained Ni, with a mean grain size of~1 μm, was also purchased from a commercial source.

### As-annealed coarse-grained Ni and ultrafine-grained Ni via ECAP

Commercial Ni rods with a diameter of 16 mm were annealed at 1073 K for 2 h to obtain a homogeneous coarse-grained (CG) microstructure with a mean grain size of 35 μm. The ECAP processing was carried out using a 16-mm diameter die with an intersecting channel angle of 90° and an outer arc angle of 45°. This die configuration imposes an effective strain of approximately one per ECAP pass.

### Tensile tests

Quasi-static uniaxial tensile tests were carriedout using an Instron 5582 (or 5966) testing machine at a strain rate of 5 × 10^−4^ s^−1^ at room temperature. A laser extensometer, P-50 by Fiedler Optoelectronics, was used to measure tensile strains for nanodomained Ni. The gauge section had a length of 10 mm, width of 2.5 mm, and thickness of ~100 μm. Six tensile tests were conducted for the purpose of providing repeatable and convincing mechanical property. The first four tests were for two tensile samples in each batch. About eight month later, two more tests were added. All the tensile engineering stress *vs* strain curves are shown in Fig. S10 in Supporting Information, showing consistent properties.

For other tensile samples, the gauge section dimensions were 10 mm × 2.5 mm × 150 μm for ED-NC Ni, 10 mm × 2.5 mm × 100 μm for ED-UFGNi, and 10 mm × 2.5 mm × 1 mm for ED-Ni, ECAP-Ni, and CG-Ni. An extensometer was used to measure strain during uniform tensile elongation for these samples. In order to confirm the reproducibility of the tensile property, for each type of Ni tensile tests were carried out at least 3 to 5 times at a strain rate of 5 × 10^−4^ s^−1^ at room temperature.

### Transmission electron microscopy

The lattice images of dislocation patterns and distribution of domain sizes were obtained using a high-resolution electron microscope (HREM), JEM2100F (JOEL Ltd., Tokyo, Japan) operating at 200 kV. The diffraction contrast images of dislocation behaviors were acquired using a conventional transmission electron microscope (TEM), Tecnai G2 20 (FEI Corporation, Netherlands). TEM specimens for ex-situ observations were cut from the gauge section of the tensile sample, and prepared by conventional twin-jet polishing technique using a nitric acid–methanol solution (20% by volume of HNO3) at −30 °C.

### X-ray diffraction

X-ray diffraction (XRD) measurements were performed with a Scintag X-ray diffracto- meter, using a Cu target operating at 1.8 kW and a graphite curved single crystal 〈0002〉 monochromator to select the Cu Kα radiation.

### MD simulations of dislocation interactions with nanodomains

The MD simulations were carried out using the Large-scale Atomic/Molecular Massively Parallel Simulator (LAMMPS) code and a Ni EAM potential^44^. The interaction of a straight edge dislocation with nanodomains was simulated in a cell (Fig. 5a) with dimensions of 69.69 × 20.73 × 41.39 nm^3^ (5.5 million atoms). The fcc Ni lattice was bounded by 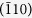, 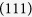 and 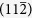 faces in the X, Y and Z directions, respectively. The continuum displacement field of an edge dislocation was used to create a dislocation with Burgers vector of 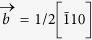 in the center of the simulation cell (X = Y = 0) with a line direction parallel to the Z axis. Two nanodomains (diameter = 7.2 nm) separated by a distance of 20.7 nm (half length along Z axis) were created by rotating the corresponding spherical lattices about the X axis for 6° (LAGB domains) or 90° (HAGB domains). Periodic boundary conditions were applied in the Z direction, whereas X direction was set to be free. In Y direction, the cell was divided into three regions consisting of a freely mobile block and two thin rigid blocks (top and bottom of the box) wherein the atoms are fixed in their positions. Before shear loading, the as-created samples were first subjected to energy minimization using the conjugate gradient method, then gradually heated up to the desired temperature in a step-wise fashion, and finally relaxed for 100 ps in the Nose/Hoover isobaric-isothermal ensemble (NPT) under zero pressure and 1 K. The cell was then loaded in shear by subjecting the atoms in the top and bottom rigid blocks to a constant velocity in the X direction at a shear strain rate of 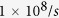. Common neighbor analysis (CNA) was used to differential atoms in different environments, gray color for perfect fcc, red for hcp and green for those at grain boundaries, dislocation core, and free surface.

## Additional Information

**How to cite this article**: Wu, X. *et al.* Nanodomained Nickel Unite Nanocrystal Strength with Coarse-Grain Ductility. *Sci. Rep.* **5**, 11728; doi: 10.1038/srep11728 (2015).

## Supplementary Material

Supplementary Information

## References

[b1] Lu K., Lu L. & Suresh S. Strengthening materials by engineering coherent internal boundaries at the nanoscale. Science 324, 349–352 (2009). 19372422 10.1126/science.1159610

[b2] Jang D., Li X., Gao H. & Greer J. R. Deformation mechanisms in nanotwinned metal nanopillars. Nature Nanotechnol 7, 594–601 (2012). 22796745 10.1038/nnano.2012.116

[b3] Wang Y. M., Chen M. W., Zhou F. H. & Ma E. High tensile ductility in a nanostructured metal. Nature 419, 912–915 (2002). 12410306 10.1038/nature01133

[b4] Lu L., Shen Y., Chen X., Qian L. & Lu K. Ultrahigh strength and high electrical conductivity in copper. Science 304, 422–426 (2004). 15031435 10.1126/science.1092905

[b5] Youssef K. M., Scattergood R. O., Murty K. L., Horton J. A. & Koch C. C. Ultrahigh strength and high ductility of bulk nanocrystalline copper. Appl. Phys. Lett. 87, 091904 (2005).

[b6] Valiev R. Z., Alexandrova I. V., Zhu Y. T. & Lowe T. C. Paradox of strength and ductility in metals processed by severe plastic deformation. J. Mater. Res. 17, 5–8 (2002).

[b7] Zhao Y. H. *et al.* High tensile ductility and strength in bulk nanostructured nickel. Adv. Mater. 20, 3028–3033 (2008).

[b8] Fang T. H., Li W. L., Tao N. R. & Lu K. Revealing extraordinary intrinsic tensile plasticity in gradient nano-grained copper. Science 331, 1587–1590 (2011). 21330487 10.1126/science.1200177

[b9] Wu X. L., Jiang P., Chen L., Yuan F. P. & Zhu Y. T. Extraordinary strain hardening by gradient structure. Proc. Natl. Acad. Sci. USA 111, 7197–7201 (2014). 24799688 10.1073/pnas.1324069111 PMC4034219

[b10] Wei Y. J. *et al.* Evading the strength–ductility trade-off dilemma in steel through gradient hierarchical nanotwins. Nature Commun. 5, 3580 (2014). 24686581 10.1038/ncomms4580 PMC3988817

[b11] Huang X. X., Hansen N. & Tsuji N. Hardening by annealing and softening by deformation in nanostructured metals. Science 312, 249–251 (2006). 16614217 10.1126/science.1124268

[b12] Valiev R. Z. Nanostructuring of metals by severe plastic deformation for advanced properties. Nature Mater. 3, 511–516 (2004). 15286754 10.1038/nmat1180

[b13] Zhu Y. T. & Liao X. Z. Nanostructured metals-retaining ductility. Nature Mater. 3, 351–352 (2004). 15173850 10.1038/nmat1141

[b14] Ma E. Eight routes to improve the tensile ductility of bulk nanostructured metals and alloys. JOM 58, 49–53 (2006).

[b15] Zhu Y. T. & Langdon T. G. The fundamentals of nanostructured materials processed by severe plastic deformation. JOM 56, 58–63 (2004).

[b16] Meyers M. A., Mishra A. & Benson D. J. Mechanical properties of nanocrystalline materials. Prog. Mater. Sci. 51, 427–556 (2006).

[b17] Dalla T. F., Spatzig P., Schaublin R. & Victoria M. Deformation behaviour and microstructure of nanocrystalline electrodeposited and high pressure torsioned nickel. Acta Mater. 53, 2337–2349 (2005).

[b18] Krasilnikov N., Lojkowski W., Pakiela Z. & Valiev R. Z. Tensile strength and ductility of ultra-fine-grained nickel processed by severe plastic deformation. Mater. Sci. Eng. A 397, 330–337 (2005).

[b19] Schwaiger R., Moser B., Dao M., Chollacoop N. & Suresh S. Some critical experiments on the strain-rate sensitivity of nanocrystalline nickel. Acta Mater. 51, 5159–5172 (2003).

[b20] Gu C. D., Lian J. S. & Jiang Q. Layered nanostructured Ni with modulated hardness fabricated by surfactant-assistant electrodeposition. Scr. Mater. 57, 233–236 (2007).

[b21] Erb U., Sherik A. M. El., Palumbo G. & Aust K. T. Synthesis, structure and properties of electroplated nanocrystalline materials. Nanostruct. Mater. 2, 383–390 (1993).

[b22] Torre F. D., Swygenhoven H., Van & Victoria M. Nanocrystalline electrodeposited Ni: microstructure and tensile properties. Acta Mater. 50, 3957–3970 (2002).

[b23] Liu B. *et al.* Dislocation interactions and low-angle grain boundary strengthening. Acta Mater. 59, 7125–7134 (2011).

[b24] Liu B., Eisenlohr P., Roters F. & Raabe D. Simulation of dislocation penetration through a general low-angle grain boundary. Acta Mater. 60, 5380–5390 (2012).

[b25] Shen Z., Wagoner R. H. & Clark W. A. T. Dislocation and grain boundary interactions in metals. Acta Metall. 36, 3231–3242 (1988).

[b26] Dingley D. J. & Pond R. C. On the interaction of crystal dislocations with grain boundaries. Acta Metall. 27, 667–682 (1979).

[b27] Sangid M. D., Ezaz T., Sehitoglu H. & Robertson I. M. Energy of slip transmission and nucleation at grain boundaries. Acta Mater. 59, 283–296 (2011).

[b28] Koning M., de, Miller R., Bulatov V. V. & Abraham F. F. Modeling grain-boundary resistance in intergranular dislocation slip transmission. Phil. Mag. A 82, 2511–2527 (2002).

[b29] Farkas D. & Patrick L. Tensile deformation of fcc Ni as described by an EAM potential. Phil. Mag. 89, 3435–3450 (2009).

[b30] Li J. C. M. Mechanical grain growth in nanocrystalline copper. Phys. Rev. Lett. 96, 215506 (2006). 16803250 10.1103/PhysRevLett.96.215506

[b31] Wang L. H. *et al.* Grain Rotation Mediated by Grain Boundary Dislocations: Atomic-Scale Quantitative Observations. Nature Commun. 5, 4402. (2014). 25030380 10.1038/ncomms5402 PMC4109021

[b32] Liu X. C., Zhang H. W. & Lu K. Strain-induced ultrahard and ultrastable nanolaminated structure in nickel. Science 342, 337–340 (2013). 24136963 10.1126/science.1242578

[b33] Lim A. T., Srolovitz D. J. & Haataja M. Low-angle grain boundary migration in the presence of extrinsic dislocations. Acta Mater. 57, 5013–5022 (2009).

[b34] Rupert T. J. & Schuh C. A. Mechanically driven grain boundary relaxation: a mechanism for cyclic hardening in nanocrystalline Ni. Phil. Mag. Lett. 92, 20–28 (2012).

[b35] Caturla M. J., Nieh T. G. & Stolken J. S. Differences in deformation processes in nanocrystalline nickel with low-and high-angle boundaries from atomistic simulations. Appl Phys Lett 84, 598–600 (2004).

[b36] Ashby M. F. in Oxide Dispersion Strengthening, Ansell G. S. , ed. (New York: Gordon and Breach), pp. 431–468 (1968).

[b37] da Costa Teixeira J., Bourgeois L., Sinclair C. W. & Hutchinson C. R. The effect of shear-resistant, plate-shaped precipitates on the work hardening of Al alloys: Towards a prediction of the strength-elongation correlation. Acta Mater. 57, 6075–6089 (2009).

[b38] Zhao Y. H., Liao X. Z., Cheng S., Ma E. & Zhu Y. T. Simultaneously increasing the ductility and strength of nanostructured alloys. Adv Mater. 18, 2280–2283 (2006).

[b39] Cheng S., Zhao Y. H., Zhu Y. T. & Ma E. Optimizing the strength and ductility of fine structured 2024 Al alloy by nano-precipitation. Acta Mater. 55, 5822–5832 (2007).

[b40] Liddicoat P. V. *et al.* Nanostructural hierarchy increases the strength of aluminium alloys, Nature Commun. 1, 63 (2010). 20842199 10.1038/ncomms1062

[b41] Liu G. *et al.* Nanostructured high-strength molybdenum alloys with unprecedented tensile ductility. Nature Mater. 12, 344–350 (2013). 23353630 10.1038/nmat3544

[b42] Wu X. L., Zhu Y. T., Wei Y. G. & Wei Q. Strong Strain Hardening in Nanocrystalline Nickel. Phys. Rev. Lett. 103, 205504 (2009). 20365992 10.1103/PhysRevLett.103.205504

[b43] Swygenhoven H. van., Derlet P. M. & Frøseth A. G. Stacking fault energies and slip in nanocrystalline metals. Nature Mater. 3, 399–403 (2004). 15156199 10.1038/nmat1136

[b44] Mishin Y., Farkas D., Mehl M. J. & Papaconstantopoulos D. A. Interatomic potentials for monoatomic metals from experimental data and *ab initio* calculations. Phys. Rev. B 59, 3393 (1999).

