# Supplementary material for: Nanodomained Nickel Unite Nanocrystal Strength with Coarse-Grain Ductility
Source: Sci Rep. 2015 Jun 30;5:11728. doi: 10.1038/srep11728 (PMC4485168; doi:10.1038/srep11728)
Supplement: Supplementary Information [file srep11728-s1.pdf]

Nanodomained Nickel Unite Nanocrystal Strength with Coarse-Grain Ductility

Xiaolei Wu[1☆], Fuping Yuan[1], Muxin Yang[1], Ping Jiang[1], Chuanxin Zhang[1], Liu Chen[1], Yueguang Wei[1], EvanMa[2☆]

[1] State Key Laboratory of Nonlinear Mechanics, Institute of Mechanics, Chinese Academy of Sciences, Beijing 100190, China

[2] Department of Materials Science and Engineering, The Johns Hopkins University, Baltimore, Maryland 21218, USA

☆ Correspondence and requests for materials should be addressed to
xlwu@imech.ac.cn , ema@jhu.edu

Supplementary Information

Table S1. Chemical analysis of main impurity contents in as-deposited Ni sheets. (two separate batches)

Element	Co	Mn	Si	S	Al	C	H	O	N
Content (mass ppm)	465	103	228	166	88	88	31	-	-
	490	120	266	168	80	68	26	-	-

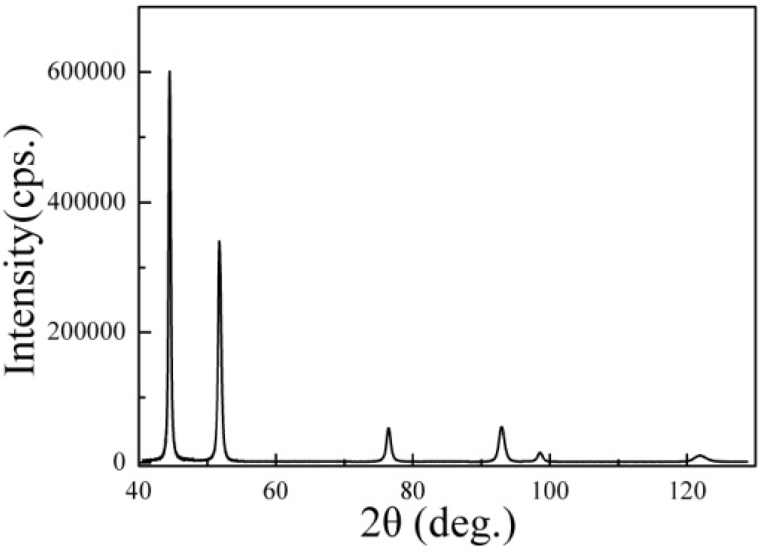

Figure S1. XRD spectrum of nanodomained Ni. The foil had a thickness of 80 μm. The relative peak intensities suggest a moderate degree of (111) texture. A cross-sectional TEM micrograph is shown in Fig. S2, showing minor columnar structure.

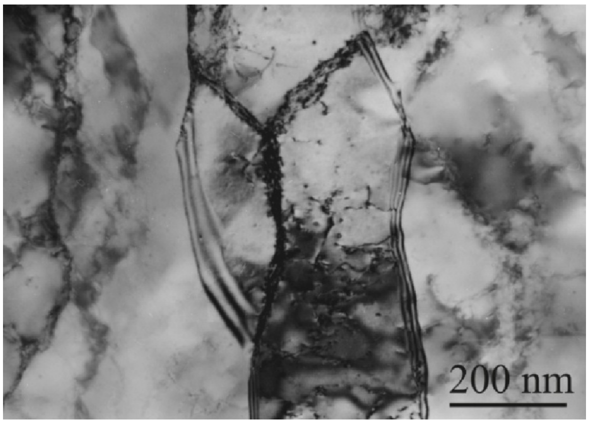

Figure S2. Cross-sectional TEM micrograph of ED UFG-Ni with nanodomains before tensile test.

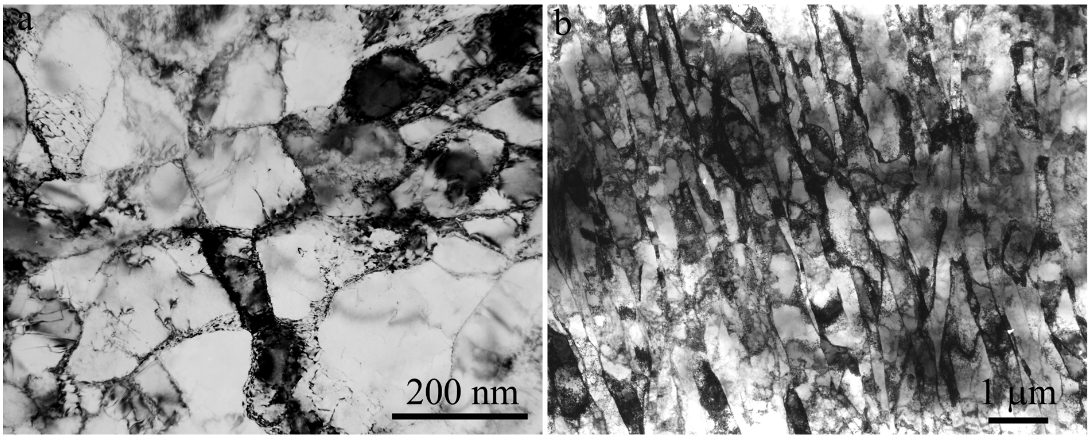

Figure S3. UFG grain structure of (a) electrodeposited fine-grained Ni and (b) ECAP-Ni, after tensile straining to 8% and 1%, respectively.

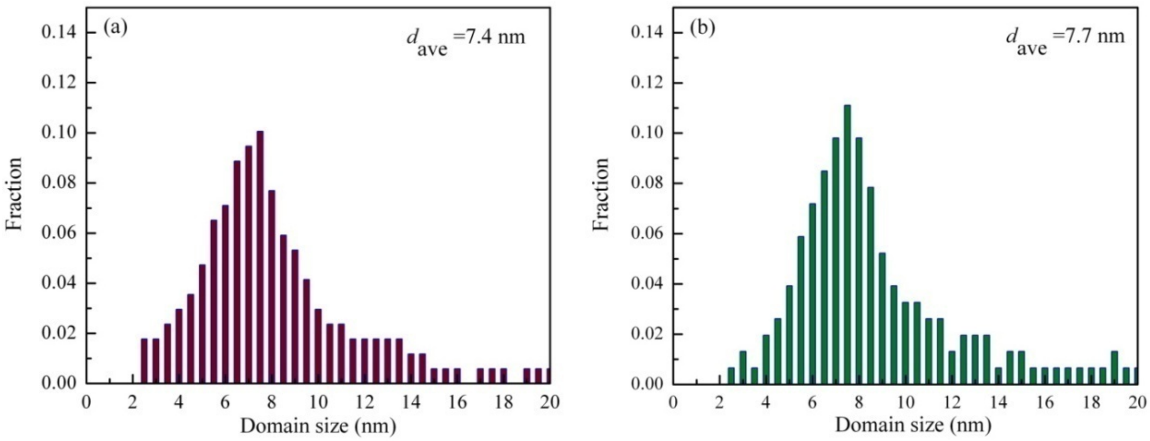

Figure S4. Size distribution of nanodomains (a) before (306 domains) and (b) after (169 domains) tensile straining to 20% elongation. The average size is about 7 nm.

The domains have an average spacing, L, of ~20 nm based on statistical spacing analysis between adjacent grains more than 100 data from TEM images. Then the volume fraction of the domains (considering spherical shape) in the sample can be obtained to be ~ 2.4% through the equation: $f = \frac{\pi d_{domain}^3}{6L^3}$.

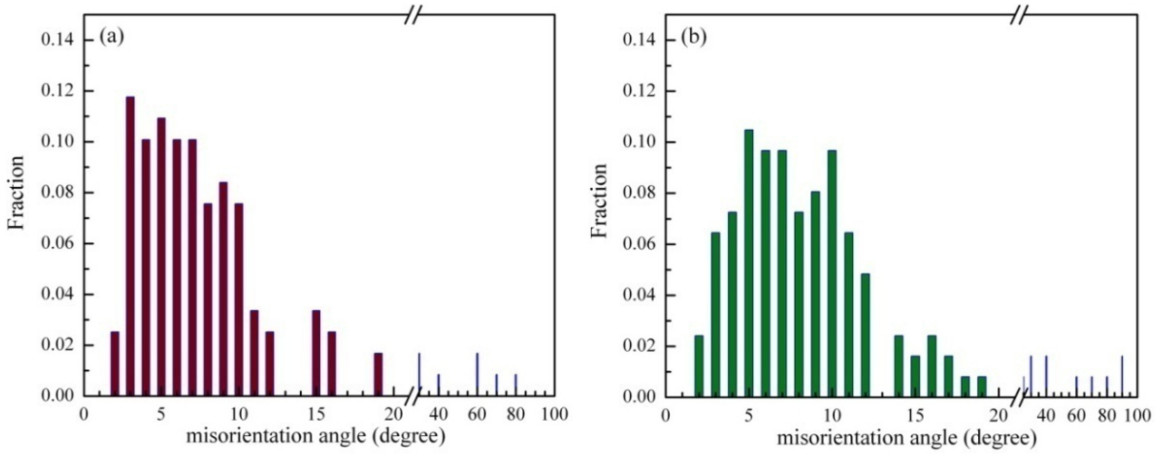

Figure S5. Statistical distribution of misorientation angle between nanodomain and surrounding matrix before (a) (306 domains) and after (b) (169 domains) tensile straining to 20 % elongation. There is no major change in the range of the angle distribution.

Prediction of nanodomain strengthening based on Ashby's model

The nanodomains act as strong obstacles to dislocation movements (much like hard particles in precipitation hardening). The dislocations therefore choose to bow around the domains, with a radius of curvature that scales with the inter-domain spacing, L, much like the scenario of Orowan strengthening. The elevation in strength (uniaxial stress) can be estimated using Ashby's model[36] for fine particles,

$$\Delta\sigma = \frac{2Gb}{2.38\pi(1-\nu)^{1/2}L}\ln\left(\frac{d_{domain}}{2b}\right) = \frac{2Gb}{2.38\pi(1-\nu)^{1/2}\sqrt[3]{\frac{\pi}{6f}}d_{domain}}\ln\left(\frac{d_{domain}}{2b}\right),$$

where d_{domain} is the average domain diameter, L is the average spacing between the domains, f is the volume fraction of the domains, G is the shear modulus (76 GPa), b is the Burgers vector (0.2489 nm), and ν is the Poisson's ratio (0.3). Fig. S6 below shows the predicted strengthening based on the equation above, as a function of f and d_{domain}. Taking d_{domain}~7 nm and f~2.4%, the predicted increase in strength is 0.8 GPa, which is in line with the strength elevation due to nanodomains observed in Figure 1.

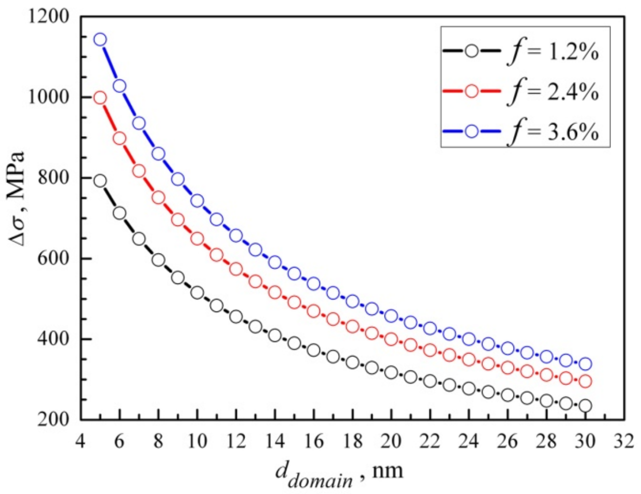

Figure S6. Dependence of nanodomain strengthening on nanodomain volume fraction (f) and domain size (d_{domain}), estimated using the Ashby model [36].

Stored dislocation density due to pinning by nanodomains

Assuming all the dislocations can be pinned by the nanodomains, the incremental rate of stored dislocation density (ρ) along with plastic straining (ε_p) can be given by [39]

$$\frac{d\rho}{d\varepsilon_p} = \frac{M}{d_{domain} b} (\frac{6f}{\pi})^{2/3}$$

where d_{domain} is the average domain diameter, f is the volume fraction of the domains, b is the Burgers vector (0.2489 nm), and M is Taylor factor with a value of 1.77. As seen from Fig. S7, the rate of stored dislocation density could be as high as $13 \times 10^{16} / m^2$ when taking d_{domain}~7 nm and f~2.4%. This is of course a crude estimate; it is meant only to make the point that the pinning and depinning motion of dislocations encountering nanodomains leads to stronger dislocation interactions and a greater likelihood of the crystal attaining a high dislocation density during deformation, giving rise to enhanced strain hardening.

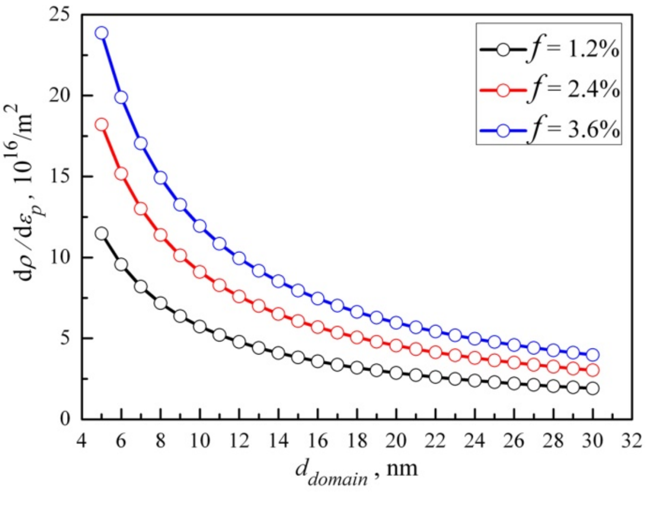

Fig. S7 Possible effect of domain volume fraction (f) and domain size (d_{domain}) on the incremental rate of stored dislocation density ($d\rho / d\varepsilon_p$).

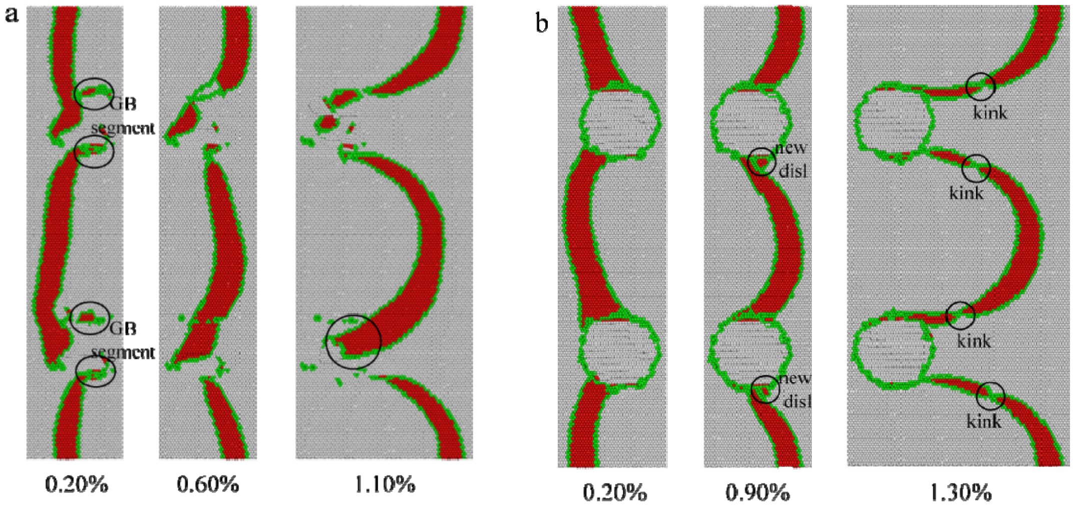

Fig. S8 A sequence of snapshots of thin slices at different shear strains showing the details of interactions between the dislocation and nano-domains: (a) LADB nanodomains; (b) HADB nanodomains.

The details for the domain boundary type effects on the interaction and pinning mechanisms will be discussed here. Thin slices along the Y direction (-0.3 nm < Y < 0.3 nm) for Figs. 5c and 5d were used to show the details of the atomistic interactions. For the low-angle domains, the boundaries are discontinuous and are split into several boundary segments due to the small misorientation angle. When the dislocation hits the low-angle domains, the dislocation is only pinned by the boundary segments and a small part of the dislocation can cut into the nano-domains. Thus the dragging force from the boundary segments should be smaller when compared to the Orowan's strengthening for "hard particles". When the dislocation hits the high-angle nano-domains, the dislocation does not cut into the nano-domains but deposits itself along and interacts with the boundary surfaces, and this mechanism is qualitatively similar to Orowan looping for "hard particles" in the textbook, resulting in higher CRSS values compared to low-angle domains. It is also observed that kinks are created in the arms of the curved dislocation by nucleation of new dislocations from boundary surfaces during the interacting process. This kink formation also helps to drag the dislocation, resulting in higher pinning strength for high-angle domains.

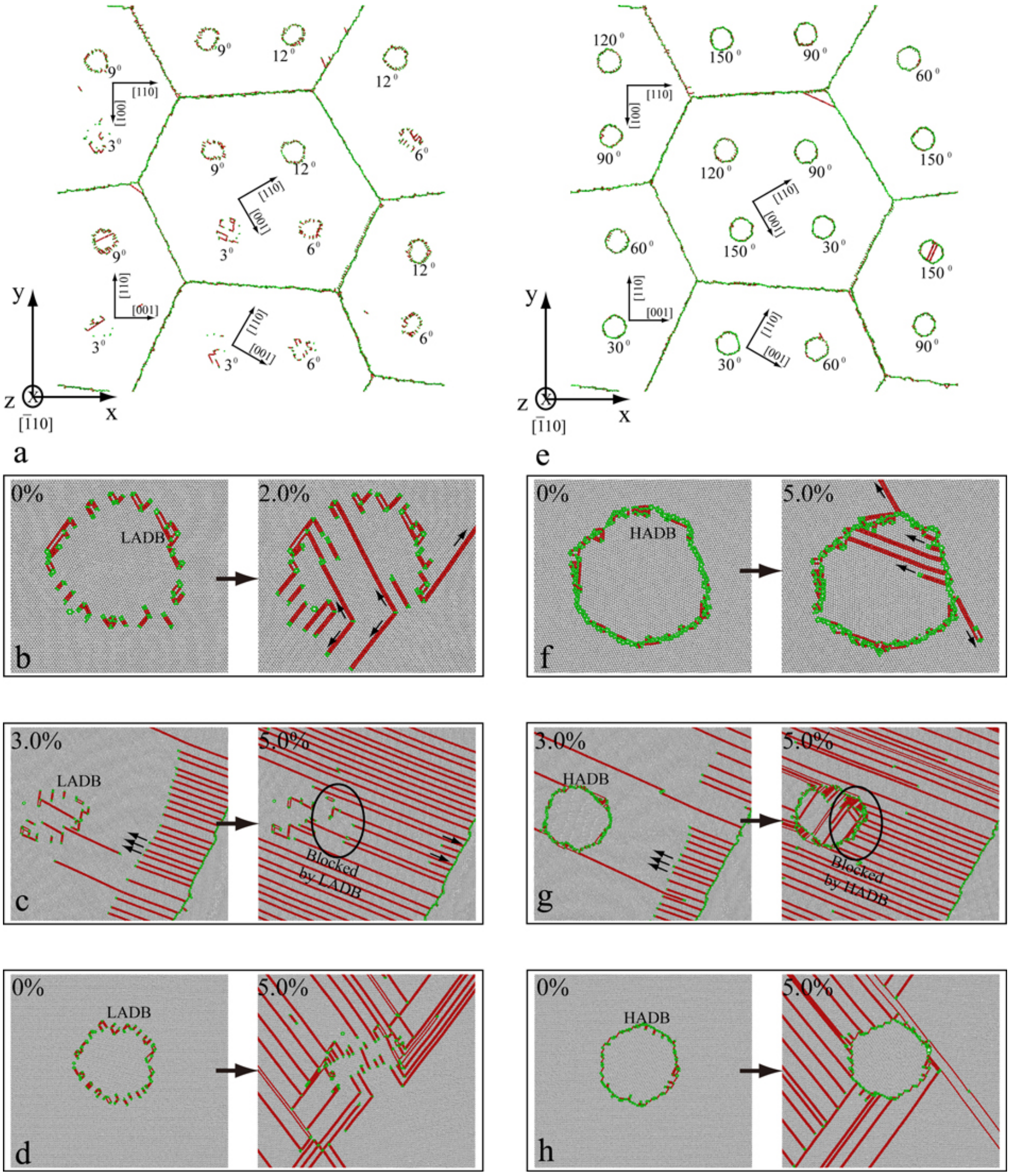

Figure S9. Relaxed columnar polycrystalline structure with (a) LADB nanodomains; (e) HADB nanodomains. The DBs of nanodomains are observed to act as sources of dislocations: (b) LADB nanodomains; (f) HADB nanodomains. The DBs of nanodomains are also observed to act as barriers of dislocations: (c) LADB nanodomains; (g) HADB nanodomains. Partial dislocations are activated in two slip systems around (d) LADB nanodomains and (h) HADB nanodomains. The corresponding tensile strain is indicated in each snapshot.

Here, we show a quasi-3-dimensional simulation with a columnar grain structure. The thickness direction contains 12 atomic planes, and is along $[\bar{1}10]$. The 4 grains were

constructed using the Voronoi method, and the average grain size was 100 nm (the sample had dimensions of $200\times200\times1.49$ nm^3, and contained approximately 5,500,000 atoms). Small Nanodomains (d_{domain}=10 nm) with LADBs or HADBs are embedded, with various misorientation angles relative to the surrounding matrix. Periodic boundary conditions were imposed along all three directions and the tensile loading was along x direction at a constant strain rate of $5\times10^8 s^{-1}$.

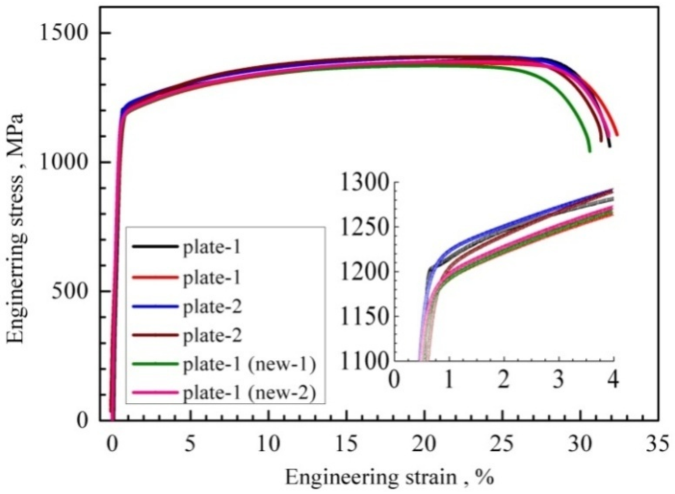

Figure S10. Tensile engineering stress-strain curves of nanodomained Ni. Six tests were conducted, on samples from two plates (batches). Note that recent tests (new-1 and new-2) for batch 1 (plate 1), after about eight months of room-temperature sample storage, indicate reproducible properties and high microstructural stability. The inset shows that the yield strength of all the tested samples/batches is within a narrow range of only ~40 MPa. Many more hardness tests were also performed, as they do not consume samples of large dimensions. The hardness results also confirm the repeatability of the properties.
